# Elastic serum-albumin based hydrogels: mechanism of formation and application in cardiac tissue engineering†

**DOI:** 10.1039/C8TB01014E

**Published:** 2018-08-23

**Authors:** Nadav Amdursky, Manuel M. Mazo, Michael R. Thomas, Eleanor J. Humphrey, Jennifer L. Puetzer, Jean-Philippe St-Pierre, Stacey C. Skaalure, Robert M. Richardson, Cesare M. Terracciano, Molly M. Stevens

**Affiliations:** a Department of Materials, Department of Bioengineering and Institute of Biomedical Engineering, Imperial College Lodon, London, SW7 2AZ, UK; b National Heart and Lung Institute, Imperial Centre for Translational and Experimental Medicine, Imperial College London, London, SW7 2AZ, UK; c H H Wills Physics Laboratory, University of Bristol, Bristol, BS8 1TL, UK

## Abstract

Hydrogels are promising materials for mimicking the extra-cellular environment. Here, we present a simple methodology for the formation of a free-standing viscoelastic hydrogel from the abundant and low cost protein serum albumin. We show that the mechanical properties of the hydrogel exhibit a complicated behaviour as a function of the weight fraction of the protein component. We further use X-ray scattering to shed light on the mechanism of gelation from the formation of a fibrillary network at low weight fractions to interconnected aggregates at higher weight fractions. Given the match between our hydrogel elasticity and that of the myocardium, we investigated its potential for supporting cardiac cells *in vitro*. Interestingly, these hydrogels support the formation of several layers of myocytes and significantly promote the maintenance of a native-like gene expression profile compared to those cultured on glass. When confronted with a multicellular ventricular cell preparation, the hydrogels can support macroscopically contracting cardiac-like tissues with a distinct cell arrangement, and form mm-long vascular-like structures. We envisage that our simple approach for the formation of an elastic substrate from an abundant protein makes the hydrogel a compelling biomedical material candidate for a wide range of cell types.

## 1 Introduction

The study of cells and tissues has long relied on traditional methods of *in vitro* culture, based on the best possible recapitulation of the cellular physiological conditions in term of nutrients and controlled environment. However, the biophysical setting of the cells is often less considered. Cells in native environments are constantly subjected to physical stimuli,^1^–^3^ which play a critical role in the maintenance of tissue integrity and the regulation of embryogenesis and disease.^4^,^5^ Amongst these stimuli, substrate stiffness is crucially important due to its regulatory role in many vital cell processes, from stem cell differentiation^6^ to the cell’s epigenetic landscape.^7^ In recent decades, a variety of hydrogels consisting of materials such as polyacrylamide, alginate, poly(ethylene glycol) and agarose, have found use as cell culture substrates with tuneable stiffnesses.^8^–^10^ Despite this, tissue-culture plastic, a stiff and non-deformable material, is the most commonly employed substrate. Here we sought to generate a simple and inexpensive material with biologically relevant substrate stiffnesses to aid the biomedical community.

We present a straightforward method to generate a free-standing biologically-relevant serum albumin (SA) hydrogel with potential to mimic the rigidity and deformability of native tissues and demonstrate its biological significance. SA is the most abundant protein in plasma, with a concentration of 35 to 50 mg ml^−1^ in human serum.^11^ Its ease of isolation, together with its abundance makes SA one of the least expensive commercially available proteins. Reports documenting the use of SA as a material for cell scaffolding have recently started to emerge,^12^–^15^ although these use enzymes for scaffold formation,^12^ requiring complicated experimental processing,^13^,^14^ or very high SA concentrations (up to 20 wt% SA),^15^ all of which constitute obstacles for more widespread use. We demonstrate a facile approach to generate free-standing hydrogels by heating an acidic solution of bovine SA (BSA) protein. Among the various available cellular systems that are sensitive to substrate modulation, we chose to work with cardiomyocytes (CMs). CMs are highly mechanosensitive cells, and their function is altered by the underlying substrate, with cells contracting more efficiently when cultured on a matrix whose stiffness approaches that of the heart.^16^ Indeed, beating of these cells is inhibited on stiff substrates that mimic a diseased (stiff) cardiac extracellular matrix (ECM).^16^,^17^ Similarly, myocardium stiffness-mimicking hydrogels can generate stem cell derived engineered cardiac tissues with an improved gene/protein expression and contraction, supplying a more mature functionality and more closely resembling their adult physiological counterpart.^18^–^21^ Here, we show that the stiffness and deformability of the engineered SA hydrogels can be easily modulated by changing the protein weight percentage, and we explore the potential of our platform to support CMs. Our results show that CMs attach and survive on the SA platform at similar levels to conventional stiffer substrates (glass), but importantly maintain a gene expression pattern similar to freshly isolated CMs. Moreover, by plating a whole ventricular cell preparation, including muscle, vascular and stromal cells, on the BSA hydrogels can generate cardiac engineered tissues with macroscopic contraction and sustained function. Cardiac cells on these engineered tissues form distinctive niches and are able to develop mm-long vessel-like structures. Taken together, we expect the method presented here to be highly accessible, and easily implemented in any laboratory environment, without the need for sophisticated equipment and synthetic protocols.

## 2 Results and discussion

### 2.1 Mechanical properties

**Fig. 1:**
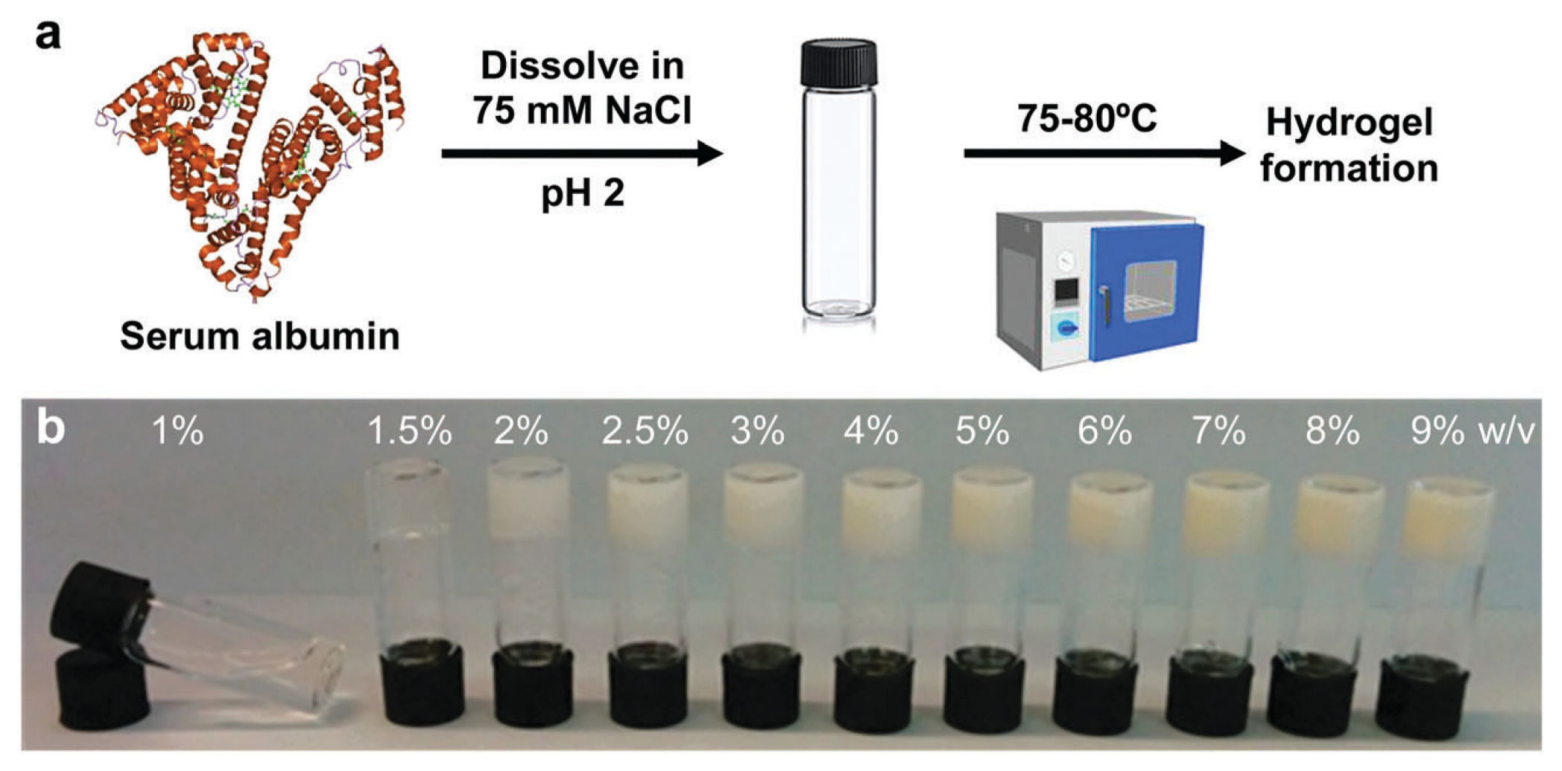
Gelation of BSA solutions. (a) Protocol for hydrogel formation. (b) Image of the BSA hydrogels at different weight fractions ≥1.5 wt% BSA, together with a solution of 1 wt% BSA that did not gel after heating.

**Fig. 2:**
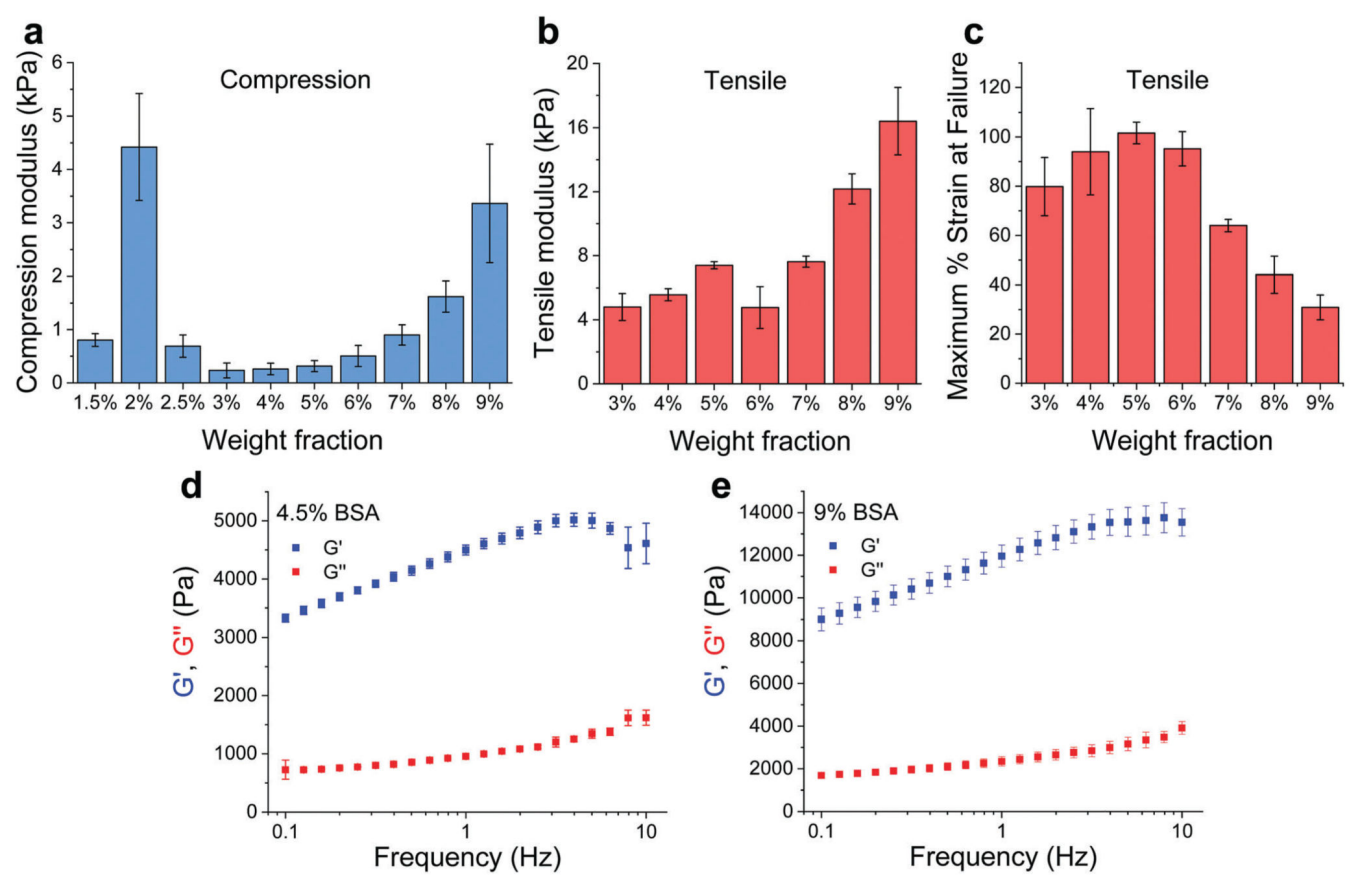
Mechanical properties of BSA hydrogels. (a) Compression (confined) and (b) tensile Young’s moduli of the BSA hydrogels at different weight fractions. (c) Maximum tensile strain percentage before failure. (d and e) Frequency-dependent rheological properties of the 4.5 and 9 wt% BSA hydrogels, respectively. All panels: mean ± SD, *n* = 3.

BSA is known to have the capacity to form fibrils.^22^,^23^ Here, we also observed that BSA could form short fibrils following heating (75 °C) of an acidic solution (pH 2, 75 mM NaCl) of 1 wt% BSA (Fig. S1, ESI†). However, we observed that further increasing the protein concentration induced a gelation process (Fig. 1a and b) in which the BSA formed a porous hydrogel (Fig. S2, ESI†). Above 1 wt% BSA (prior to heating), the protein monomer undergoes a concentration-induced structural change (from a partially-unfolded state to a molten-globule state),^24^,^25^ which correlates well with what we observe as the minimal gelation concentration. After gelation, we measured the elastic properties (Young’s modulus) of the hydrogels under confined compression (Fig. 2a) and tensile tests (Fig. 2b and c) using stress–strain analysis (Fig. S3 and S4, ESI† for compressive and tensile curves, respectively). Importantly, we found that only the ≥3 wt% BSA hydrogels could be removed intact from the template and used as free-standing self-supporting hydrogels. Accordingly, while the measurements under confined compression were possible for 1.5–9 wt% BSA hydrogels, we only performed the tensile measurements of free-standing hydrogels with the ≥3 wt% BSA hydrogels. Under compression, we found that the low weight fraction hydrogels (1.5–2.5 wt% BSA) tended to collapse (break) at ~50–70% compression strain (Fig. S3, ESI†), while hydrogels of higher weight fractions (≥3 wt% BSA) could sustain strains above 80%. We used the stress–strain curves of the confined hydrogels to calculate the compression Young’s modulus of the hydrogels, where we found an unusual concentration-dependent behaviour (Fig. 2a). At first, this Young’s modulus increased sharply to a value of 4.4 ± 1.0 kPa at 2 wt% BSA, then sharply decreased to a value of 0.2 ± 0.1 kPa at 3 wt% BSA. This was then followed by a gradual increase in the modulus as the concentration increased, where the highest fraction studied was 9% with an elastic modulus of 3.4 ± 1.1 kPa. The entire range of the compression Young’s moduli values was on the whole within the lower end of the elastic moduli for commonly used hydrogels.^26^–^28^

As stated above, the hydrogels could only be removed from their templates and serve as free-standing materials at BSA weight fractions of ≥3%. To complement our compression elasticity measurements, we also measured the tensile Young’s modulus of free-standing hydrogels (Fig. 2b). The tensile modulus of 3 to 5 wt% BSA hydrogels exhibited a slight increase as a function of concentration. However, a small drop in this modulus was observed at 6 wt% BSA, which was followed by a further increase in the tensile modulus as a function of concentration >6 wt% BSA. The strain at failure analysis (Fig. 2c) revealed that the hydrogels were highly elastic, extending to as much as twice their initial length at ≤6 wt% BSA, while at >6 wt% BSA they became more brittle and less extensible as a function of concentration. To further probe the viscoelastic properties of the hydrogels, we assessed the frequency-dependent rheological properties of the BSA hydrogels at weight concentrations of 4.5 and 9 wt% BSA (Fig. 2d and e, respectively) and at frequencies of 0.1–10 Hz, which includes the 1–3 Hz regime of the heart. These concentrations were chosen to cover the two extremes found for the BSA hydrogels, namely the lowest compression modulus with the highest tensile strain at failure *vs.* the highest compression modulus (while excluding the 2 wt% BSA hydrogel as it is not free-standing) and the lowest tensile strain at failure. These weight concentrations were also chosen for the cardiac cells scaffolds (*vide infra*). As can be seen from the graphs, the storage modulus (*G*′, elastic response) is much larger than the loss modulus (*G*″, viscous behaviour), indicating the highly elastic nature of the BSA hydrogels, and the frequency-dependence of the modulus within the frequency range of interest is indicative of a viscoelastic material.

### 2.2 Mechanism of gelation

**Fig. 3:**
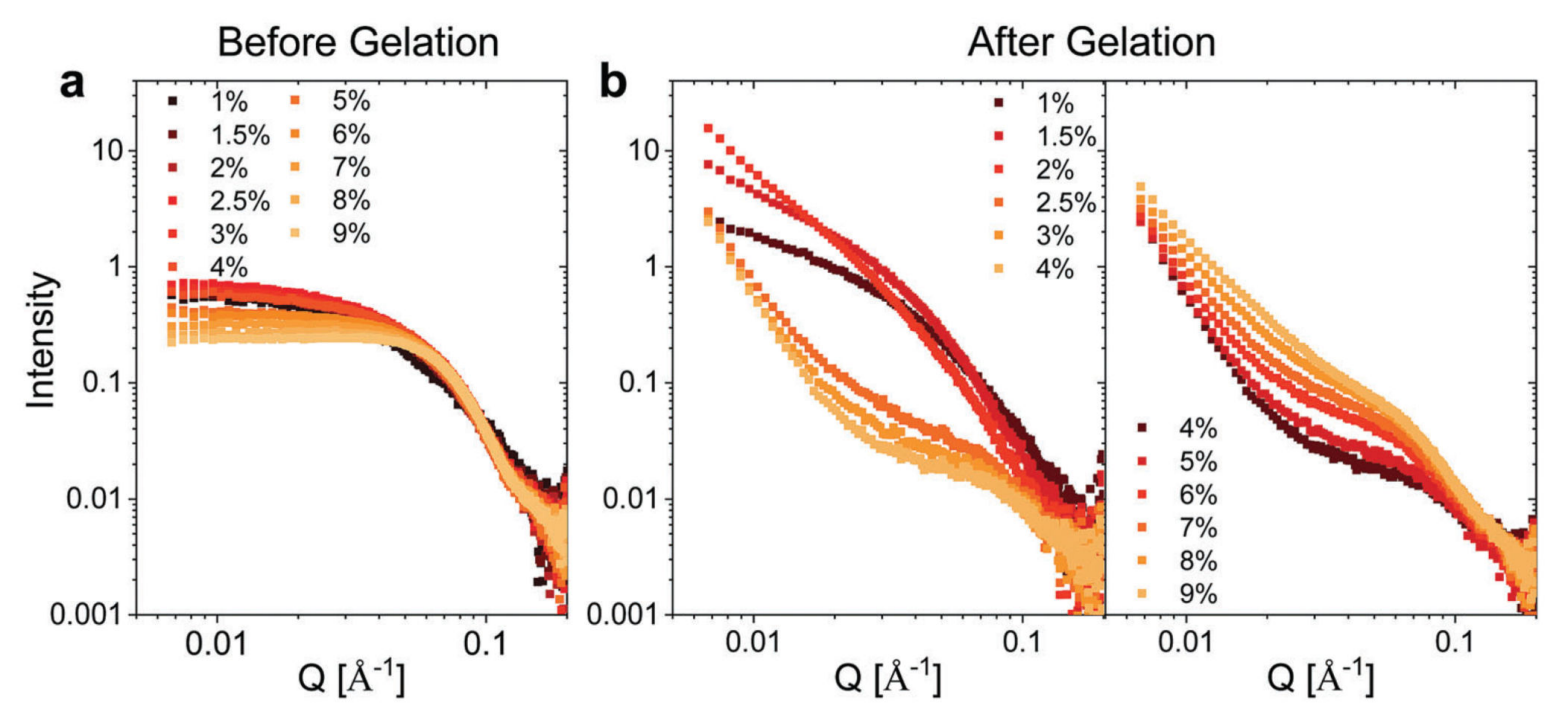
SAXS measurements. Concentration normalized SAXS intensity for the different weight fractions of BSA (a) before and (b) after gelation.

We utilized small angle X-ray scattering (SAXS) analysis to investigate whether differences in the internal structure of the hydrogels at different weight fractions could account for the unique mechanical properties observed for the hydrogels. Prior to gelation, BSA exhibits a strongly positively charged surface that is shielded by the high concentration of NaCl employed. At protein weight fractions of >3 wt% we observed a decrease in the low *Q* scattering intensity and the development of a prominent shoulder in the region of *Q* = 0.05 Å^−1^, which is suggestive of inter-protein repulsion effects (Fig. 3a). We postulate that this effect of inter-protein separation leads to a transition from a partially unfolded state of BSA to a molten-globule state with resulting changes in exposed charge.^24^ The scattering profile at ≤2 wt% BSA, where the scattering is not significantly affected by inter-protein effects, is consistent with ellipsoidal structures with semiaxes in the region of 7 × 3 × 2 nm (Fig. S5 and Table S2, ESI†).

Following heating (gelation) of the BSA solutions (Fig. 3b), the fibrillation observed by atomic force microscopy (AFM) for the 1 wt% BSA sample (Fig. S1, ESI†) was also observed by SAXS measurements, where the scattered intensity of the sample could be fitted to non-oriented, ellipsoidal cross section cylinders with diameters similar to pre-heated BSA at 1 wt% (Fig. S6 and Table S3, ESI†). Approaching 2 wt% BSA, we observed a distinct increase in low *Q* scattered intensity, indicative of structural inhomogeneity approaching the micron-scale due to aggregation of the fibrils. Above 2.5 wt% BSA, gelation resulted in scattering from structures, which suggest coalescing aggregates of branched or potentially fractally-aggregated BSA,^29^–^31^ where a strong but rapidly decaying scattering contribution begins to dominate the low *Q* scattering as the mass fraction increases. At mass fractions greater than 4 wt%, the data conform well with an empirical model for polymer aggregation (dashed line fits in Fig. S7 and Table S4, ESI†).^24^,^32^,^33^ This model provides an insight into the two key size regimes that contribute to the scattering function in the *Q*-range between 0.006 and 0.2 Å^−1^:

(1) $$I\left(Q\right)=\frac{A}{Q^{n}}+\frac{C}{1+{\left(Q\zeta_{\text{L}}\right)}^{m}}+B$$

The first term is a Porod scattering function, with *A* being its scaling factor and the *n* exponent relating to the fractal dimension of large interconnected aggregates in the gel at the lower *Q* values measured. The second component is typically dominant at high *Q* and results from the smaller inter-chain distances within the proteins themselves where, depending on the state of the gelled material, it can approximately describe the non-gelled BSA fraction or in a uniform gel this component can be used to determine a mesh size. This second term is described by a scaling factor, *C*, and a modified Lorentzian function of exponent *m* with a Lorentzian screening factor term ζ_L_, the indicator for the mesh size of the gel. Gelled samples with a mass fraction of ≥4 wt% could be effectively simulated using this model (Fig. S7a, fitting parameters in Table S3, ESI†), where the values of the exponents *n* and *m* were fixed at 3.5 and 3 respectively. This enabled the concentration-weighted contribution of the Porod and Lorentzian scaling factors *A* and *C* to be followed. At higher mass fractions, both scaling factors increased as a function of the weight fraction (Table S3, ESI†). The value of *A*/*C* (Fig. S7b, ESI†) decreased at mass fractions greater than 4 wt% suggesting a transition from a state with a sizeable fraction of larger, micron-scale aggregates with a large Porod contribution to one with a more homogeneous (*i.e.* less fractal) nanoscale gel at higher mass fractions. In summary, the use of the model suggests a transition from a state with larger micron-scale aggregates at low weight fractions to one with a more homogeneous nanoscale gel at higher weight fractions.

Altogether, the interpretation of the scattering features would suggest that the gelation of the 2 wt% BSA hydrogel is likely to occur *via* aggregation of BSA fibrils, which results in a stiffer hydrogel. Increasing the weight fraction > 2–2.5 wt% BSA resulted in the collapse of the fibrillar network and the formation of interconnected aggregates, which enabled the hydrogels to serve as an insoluble free-standing material, and also induced a decrease in the stiffness values. Increasing the weight fractions beyond 4 wt% BSA led to an increased connectivity of the structure, which in turn reduced the elasticity of the gels, making them brittle and increasing their stiffness.

### 2.3 BSA hydrogels as scaffolds for cardiomyocytes

Following the generation of a self-standing highly elastic BSA hydrogel substrate with biologically-relevant mechanical properties, we explored the interaction with CMs since the ability of these cells to develop and function (beat) effectively is significantly modulated by the elastic nature of the surrounding ECM forming the tissue.^2^ This is highlighted by the adverse changes, such as dedifferentiation, loss of gene expression and functionality, which are associated with an extensive stiffening of the CM environment and occurs in diseased myocardium.^34^,^35^ Cardiac tissue has an elasticity (stiffness) of 10–20 kPa in compression and a tensile strength of 30–70 kPa (values are for rat myocardium) along with an ability to accommodate ~20% strain, essential for effective operation of the myocardial cells as a beating ensemble.^16^,^36^ The compressive (0.25–3.3 kPa, Fig. 2b) and tensile (5–16 kPa, Fig. 2c) moduli of the free-standing (≥3 wt% BSA) BSA hydrogels together with their viscoelastic nature makes them appealing candidates for cardiac tissue engineering applications, since they should not inhibit the mechanical contraction of myocardial cells in the way that stiffer scaffolds can.^16^

**Fig. 4:**
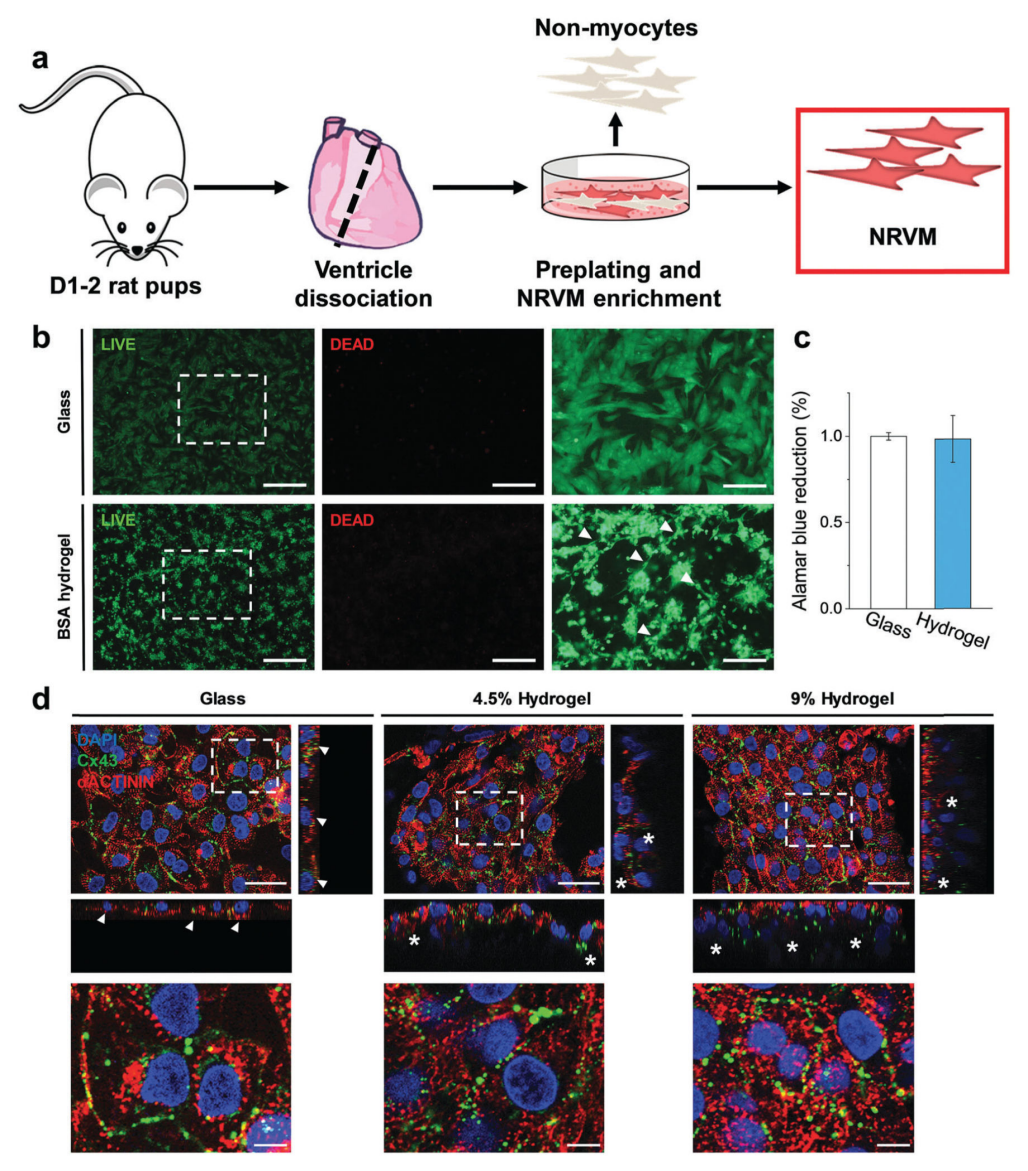
BSA hydrogels as cell scaffolds. (a) Diagram of neonatal rat ventricular CM isolation. (b) LIVE/DEAD™ staining of 500k CMs plated on glass (top) and 4.5% wt BSA hydrogel (bottom), alongside with their higher magnification images (right images). The arrowheads point to interconnected cell clusters. (c) AlamarBlue^®^ reduction on day 3 after plating on glass or on BSA hydrogels. *n* = 3, 3 biological replicates per condition. (d) Confocal imaging. Cx43 (green), α-actinin (red) and DAPI (blue) staining. The bottom images are magnifications of the top ones. The orthogonal views (narrow rectangles) show that CMs on glass form a flat monolayer (arrowheads), while BSA hydrogels support the formation of interconnected cell layers, as depicted by Cx43-interconnected layers (asterisks). Scale bars: (b) left and centre: 500 μm; right: 200 μm; (d) top: 25 μm; bottom: 5 μm.

We therefore set out to investigate the BSA hydrogels as physiologically relevant cell substrates at two different weight fractions, namely 4.5 wt% and 9 wt% BSA, in order to probe two sets of physical properties within the optimal limits of the healthy myocardium. For the CM cells, we chose to work with neonatal rat ventricular myocytes (Fig. 4a), and to evaluate their cellular responses shortly after seeding (3 days) to probe any early relevant responses from the CM. Neonatal rat ventricular myocytes readily respond to changes in substrate mechanics^17^,^19^,^20^ and, although a differentiated and functional cell type, they are still subjected to significant changes related to cell maturation after birth.^37^,^38^ We coated substrates (hydrogels and glass coverslips as controls) with fibronectin to facilitate CM attachment, as confirmed using fibronectin immunostaining (Fig. S8, ESI†). Interestingly, and even though BSA is frequently used to prevent non-specific interaction of other proteins, fibronectin adsorbed to our BSA hydrogels. We speculate that the suggested exposure of the BSA binding sites in our acidic medium^24^,^25^ induces a change in the protein’s surface charge, which facilitates protein adsorption. Indeed, we recently showed that electrospun fibrous BSA scaffolds can efficiently bind the protein laminin.^39^ LIVE/DEAD™ staining 24 hours after seeding showed low levels (<1%) of cell death, with CMs distributed throughout the surface of the BSA hydrogels (Fig. 4b), forming a network of interconnected cell clusters (Fig. 4b, arrowheads). In addition, AlamarBlue^®^ reduction on day 3 after plating showed no significant differences between cells plated on glass coverslips or on the hydrogels, thus pointing towards similar levels of engraftment (Fig. 4c). Immunofluorescent staining (Fig. 4d) revealed that both the cells on glass and on the BSA substrates expressed the gap junction protein Connexin43 (Cx43) and the cardiac specific sarcomeric protein α-actinin. However, while the cells on glass formed a flat monolayer (Fig. 4d, arrowheads), those plated on the BSA hydrogels were able to partially penetrate the material (Fig. 4d, asterisks) and connect by Cx43-positive gap junctions. In addition, all cells presented α-actinin-positive striations (Fig. 4d, bottom, and Fig. S9, ESI† for additional staining showing the striations in better resolution), with the majority of the Cx43-positive signal located at the membrane-to-membrane interface. Sarcomere length quantification (Fig. S10, ESI†) showed that cells on the hydrogels have increased Z-line-to-Z-line distance (~1.55 μm) compared to the cells on glass (1.49 μm), although this parameter was still shorter than those of the adult rat CM, which is usually around 1.7–1.8 μm.^40^

**Fig. 5:**
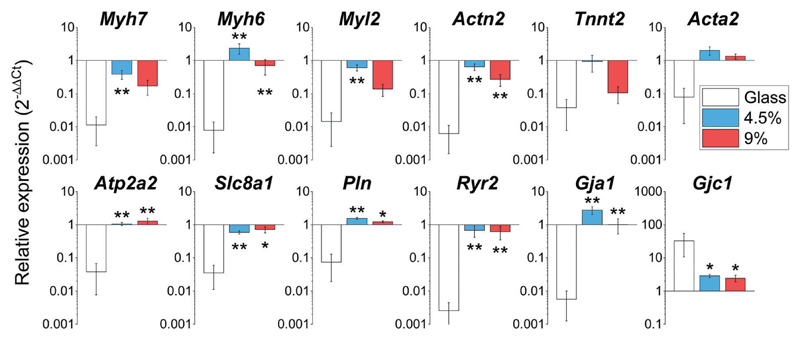
Gene expression profiling of cardiac genes on day 3 after plating on glass, 4.5 and 9 wt% BSA hydrogels. *: *p* < 0.05 *vs.* glass; **: *p* < 0.01 *vs.* glass. All graphs: mean ± SEM; *n* = 3–6 with 3 biological replicates per condition.

Rapid changes in gene expression akin to a dedifferentiation process are known to occur when CMs are plated on stiff substrates such as glass or tissue culture plastic.^16^ We profiled the expression of a wide array of cardiac genes (Fig. 5) including crucial members of the contractile apparatus (β-and α-myosin heavy chains (*Myh7* and *Myh6*), ventricular myosin light chain (*Myl2*), cardiac α-actinin (*Actn2*), cardiac troponin T (*Tnnt2*) and smooth muscle actin (*Acta2*), regulators of functionality and calcium homeostasis (SERCA2 (*Atp2a2*)), membrane sodium–calcium exchanger (*Slc8a1*), phospholamban (*Pln*) and ryanodine receptor 2 (*Ryr2*)), as well as the main cardiac connexins (Connexin 43 (*Gja1*) and 45 (*Gjc1*)). Our analysis revealed that the expression levels of these genes for cells cultured on the hydrogels were highly similar to the expression level of the freshly isolated CMs (gene expression was normalized to freshly isolated CMs in Fig. 5). By contrast, the analysed genes were significantly down-regulated in myocytes plated on glass coverslips (except for Connexin 45 (*Gjc1*), which is preferentially enriched in conduction system myocytes and fibroblasts).^41^ Overall, these results demonstrate the BSA hydrogels have the ability to preserve the native expression pattern of cardiac cells far better than glass substrates.

### 2.4 BSA hydrogels contract macroscopically and support a multicellular ventricular environment

**Fig. 6:**
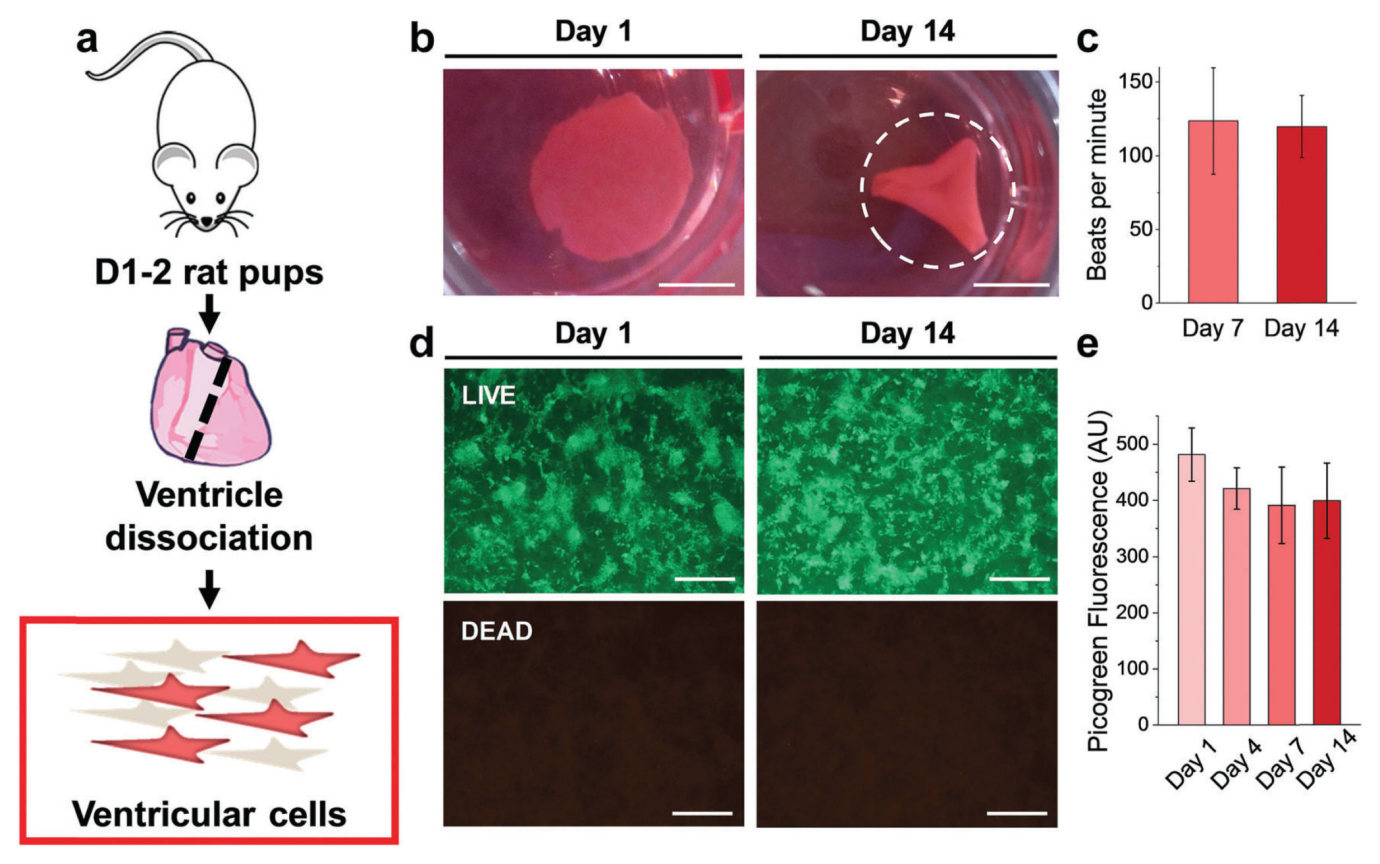
Ventricular cells on BSA substrates. (a) Diagram of ventricular cell isolation. (b) Full substrate images at days 1 and 14, showing the progressive folding of the hydrogel (dashed line representing the estimated original size of the sample). (c) Macroscopic beating rate comparison at day 7 and 14, suggesting a stable function (*p* > 0.05). (d) LIVE/DEAD™ staining of gels seeded with 500k ventricular cells at day 1 and day 14. (e) Picogreen dsDNA quantification of constructs, with no difference between time points (*p* > 0.05). Scale bars: (b) 5 mm; (d) 500 μm.

Results from the CM-only experiments show that CMs on our protein-based substrates attach and survive at similar levels as on more conventional surfaces (glass), feature a more mature sarcomeric organization and exhibit a native-like gene expression pattern. However, a better recapitulation of the multicellular environment present in the cardiac tissue must include heterocellular interactions in the myocardium and in the formation of engineered tissues, which, most importantly, can contract macroscopically.^42^ Accordingly, we performed experiments where all the cells isolated during the digestion of the ventricles (Fig. 6a) were plated on the gels, comprising not only CMs but also vascular (endothelial and smooth muscle) and stromal cells (fibroblasts). Additionally, the duration was extended to 14 days to assess any response well beyond the initial interaction, and thin 500 μm hydrogels were used. Such a reduction in gel thickness acted to decrease the mechanical resistance against the CM contraction compared to a thick substrate in these free standing gels, especially since the CMs do not significantly penetrate the substrate.

As early as 2 days after plating, strong regional contraction was noticeable even at low magnification (ESI,† Video S1). Calcein AM staining showed an extensive network of elongated cells beating in synchrony (ESI,† Video S2), indicating the capacity of electromechanical coupling between the cells. By days 4–5 post-plating, macroscopic rhythmic contraction was evident to the naked eye (ESI,† Video S3), which also led to the progressive folding of the hydrogels (Fig. 6b), likely due to the increased tension supplied by the grafted cells. This timeline of the appearance of macroscopic beating is in good agreement with recent reports for other engineered constructs.^20^,^43^ Importantly, constructs could be paced by external electrical stimulation, as shown on ESI,† Video S4, at frequencies between 1–3 Hz. The spontaneous beating rate was stable for the duration of the experiments (14 days, Fig. 6c), thus suggesting stable function of these engineered cardiac tissues. LIVE/DEAD™ staining (Fig. 6d) showed a very similar pattern of engraftment as seen in the CM-only experiments, with an interconnected network of homogeneously distributed clusters of cells. Very low levels of cell death were found, with Picogreen dsDNA quantification revealing only a slight and non-significant (*p* > 0.05) decrease in cell engraftment from day 1 to day 4.

**Fig. 7:**
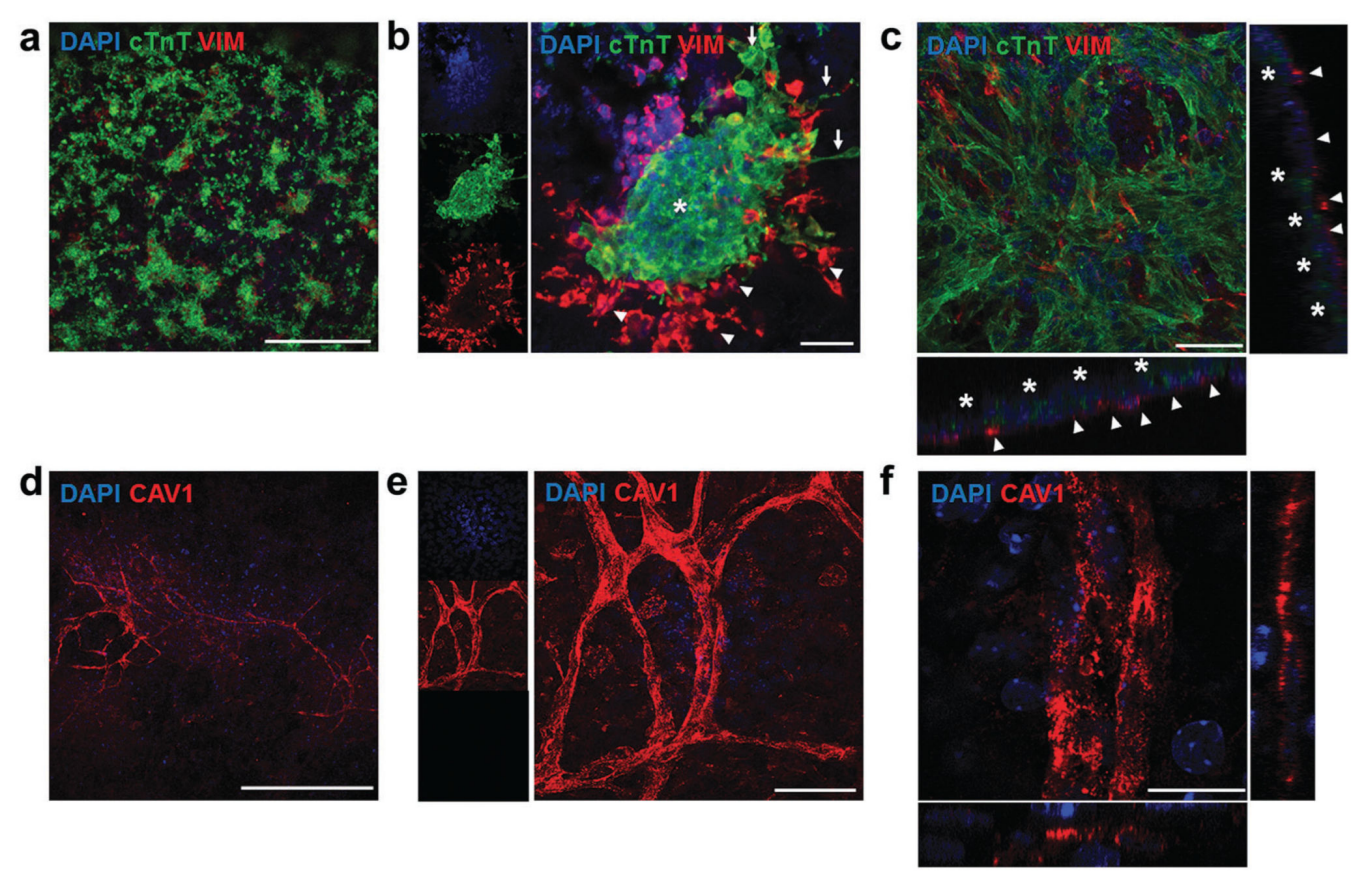
Confocal immunofluorescent staining of engineered cardiac tissues. (a) Staining for cardiac (cTnT) and stromal (Vim) markers, showed an even distribution of cell clusters on the substrate surface. (b) These structures are composed of core and inter-cluster CM bridges (asterisk and arrows respectively) and are surrounded by Vim-positive stromal cells, which are also found underneath the CMs (c, orthogonal views: arrowheads point to stromal cells while asterisks highlight cTnT ± CMs). (d–f) Confocal imaging of vessel-like structures on the engineered cardiac tissues, where Cav1 ± vascular cells form a mm-long highly branched and interconnected network (d, e for a higher magnification), but with no closed lumens (f, orthogonal views). Scale bars: a, d: 500 μm; b, c, e: 50 μm; f: 15 μm.

We further used immunostaining of fixed samples for analysing the cell distribution in our substrates. Confocal imaging for cardiac troponin T (cTnT, specifically expressed in CMs) and vimentin (Vim, a marker for stromal cells) staining revealed that CMs and stromal cells were homogeneously distributed on the hydrogel (Fig. 7a). Upon closer inspection, we found that cell clusters constituted distinct cardiac niches, where CMs formed the core as well as the cellular bridges between different clusters (Fig. 7b, asterisk and arrows respectively), thus explaining the synchronous macroscopic contraction of our engineered tissues. Vim-positive stromal cells surrounded the CMs (Fig. 7b, arrowheads) and also resided beneath the CMs (Fig. 7c, orthogonal views). Furthermore, CMs showed typical striations (Fig. S11, ESI†). Finally, staining for vascular marker caveolin-1 (Cav1) unveiled mm-long interconnected and branched structures (Fig. 7d–f), closely resembling vessels. However, upon closer inspection (Fig. 7f, orthogonal views) we were not able to identify closed lumens on these structures. Whether this is due to the lack of relevant physiological conditions, such as fluid shear flow,^44^ remains an open question to be answered in future experiments. In summary, seeding a multicellular preparation representative of the natural constituents of the cardiac tissue renders a specific cell distribution on the hydrogel, where CMs constitute a well-coupled and functional network, supported by stromal cells. Importantly, vascular cells were spontaneously able to form interconnected vessel-like structures. This organization remains stable for at least 14 days, supplying a steady function (stable beating).

## 3 Conclusion

In summary, we showed that BSA can form highly elastic free-standing hydrogels at relatively low weight fractions (>3 wt% BSA). We further revealed that subtle changes in the weight fraction of the formed hydrogel resulted in a non-linear effect on the compressive and tensile mechanical properties of the hydrogels. By using SAXS, we attributed these changes to the mechanism of hydrogel formation, which occurs due to the formation of elongated fibrils and their collapse at higher weight fractions. In this work, we focused on the possible use of the BSA hydrogels as substrates for cardiac cells. The BSA gels are biocompatible, support the attachment and spreading of CMs, as well as the development of a physiological cytoskeleton and Cx43-cellular connections. CMs on the elastic BSA hydrogels maintained a gene expression profile of crucial cardiac-related genes similar to their native signature, thereby avoiding the dedifferentiation observed on stiffer glass substrates. Using a multicellular ventricular cell preparation we were able to consistently obtain macroscopically beating engineered cardiac-like tissues. These constructs can stably function for at least 14 days, and are amenable to electrical and mechanical stimulation due to their highly elastic and free-standing nature. Our hydrogels promoted a distinct cell distribution, where CM clusters are electrically connected and at the same time supported by surrounding stromal cells. Finally, vascular cells can generate mm-long vessel-like structures on these low-stiffness substrates. The use of the developed method to form (potential autologous) SA substrates through a simple, cheap, easy-to-implement approach has the potential to find widespread applicability in biomedical applications as well as a new model system to modulate substrate mechanics.

## Supplementary Material

† Electronic supplementary information (ESI) available. See DOI: 10.1039/c8tb01014e

Supplementary information
