# Supplementary material for: Elastic serum-albumin based hydrogels: mechanism of formation and application in cardiac tissue engineering†
Source: J Mater Chem B. Author manuscript; Available in PMC 2018 Oct 1. (PMC6166857; doi:10.1039/C8TB01014E)
Supplement: Supplementary information [file NIHMS79422-supplement-Supplementary_information.pdf]

Supplementary Information for:

Elastic Serum-Albumin Based Hydrogels: Mechanism of Formation and Application in Cardiac Tissue Engineering

Nadav Amdursky,[1,†] Manuel M. Mazo,[1,†] Michael R. Thomas,[1] Eleanor Humphrey,[2] Jennifer L. Puetzer,[1] Jean-Philippe St-Pierre,[1] Stacey C. Skaalure,[1] Robert M. Richardson,[3] Cesare M. Terracciano,[2] and Molly M. Stevens[1,*]

[1]Department of Materials, Department of Bioengineering and Institute of Biomedical Engineering; [2]National Heart and Lung Institute, Imperial Centre for Translational and Experimental Medicine, Imperial College London, London, SW7 2AZ, UK; [3]H H Wills Physics Laboratory, University of Bristol, Bristol, BS8 1TL, UK.

Materials and Methods

Hydrogel preparation – Bovine serum albumin (BSA, Sigma-Aldrich, Gillingham, UK) was used for the formation of the hydrogels. The protein was dissolved in 75 mM NaCl, pH 2, at the desired weight concentration (w/v). The solution was cast to a desired container and was placed in 75°-80°C for ~ 5 hours.

Mechanical compression measurements – Volumes of 3.4 ml of BSA at concentrations ranging from 1.5 to 9 wt% BSA were prepared in tissue-culture petri dishes characterized by an inner diameter of 2 cm to form hydrogels according to the protocol described above. The hydrogels were not submerged in solution during the measurement. The height of each hydrogel was measured prior to mechanical testing. Load-displacement curves were measured in confined compression using an Instron Model 5540 testing machine (Norwood, MA, USA). A 50 N load cell was used with an indenter of 1 cm in diameter operated at a crosshead speed of 2 mm/minute, which resulted in a strain rate of ~0.4 %/second, up to a confined strain of ~80%. The Young's modulus was calculated as the slope of the stress-strain curve around the value of 5% strain (±2.5%).

Mechanical tensile measurements – 3 ml of BSA hydrogels at concentrations of 3-9 wt% BSA were prepared in a 6-well tissue-culture plate with well diameter of 3.48 cm. Following gelation, the hydrogels were removed from the well-plate and were cut into rectangles with dimensions of ~5-7 mm wide by ~8-11 mm in length. The height of each sample was measured and found to range from 1-1.6 mm. Mechanical tests were performed on an ElectroForce 3200 System (Bose, Eden Prairie, MN, USA) equipped with a 250 g load cell. Scaffolds were stretched to a preloaded of 0.03 g, allowed to relax for 60 seconds and then loaded to failure at a strain rate of 0.1 %/second, assuming quasi-static loading and ensuring failure between the grips. The reported Young's modulus was calculated based on the slope of the most linear region of stress-strain curve with a linear regressive fit of > 0.999.

Rheological measurements – 4.5% and 9 wt.% BSA hydrogels were prepared in a 6-well tissue culture plate, gelled for 5 hours at 75°C, then removed and allowed to swell to equilibrium in excess PBS overnight. After swelling, a biopsy punch was used to cut the gels into 8 mm diameter discs (n = 3 per condition). The mechanical properties of the hydrogels were studied using oscillatory shear rheology. Oscillatory parallel plate rheological measurements were performed using an Advanced Rheometer AR2000ex with AR Instrument Software (TA instruments, Herts, UK) fitted with a Peltier temperature control system. Samples were tested at 25°C using an 8 mm diameter parallel stainless steel plate. Frequency sweeps were conducted from 0.1 to 10 Hz, at 0.5% strain. For 9% gels, a normal load of 0.15 - 0.2 N was applied during testing, and for 4.5% gels, a normal load of 0.05 N was applied during testing.

Small Angle X-ray Scattering (SAXS) measurements – Non-gelled samples were investigated by adding 300 µl of BSA in the above solutions in the concentrations of 1-9 wt.% BSA into 1.5 mm quartz glass capillaries and sealed with rapid-setting epoxy resin. Scattered X-ray intensity as a function of scattering vector *Q* in the range of 0.006-0.2 $Å^{-1}$ was obtained using a CuKα Microfocus source GANESHA 300 XL (SAXSLAB, Copenhagen, Denmark) SAXS-WAXS system in SAXS mode using the in-vacuum Pilatus 300k (Dectris, Baden, Switzerland) detector. Measurements were performed following determination of the centre of the capillary by beam-stop diode measurements. Capillary contributions to scattered intensity were determined by measuring the region above the sample and a solvent subtraction was performed using 75 mM NaCl at pH 2. Following subtraction of solvent and capillary contributions considering run time and beam intensity, the two dimensional data sets were integrated azimuthally to give one-dimensional scattering profiles. Following initial measurement, the samples were removed and gelled at 80 °C for 3 hours to ensure complete gelation. The capillaries were then re-introduced to the SAXSLAB system and measurements repeated.

***Data Fitting**:* All data was fitted using the software SasView with intensity error weighting of the data over a *Q*-range of 0.0065 to 0.18 $Å^{-1}$ with the Levenberg-Marquardt algorithm to minimise $\chi^2/ Npts$. All parameters (except where referred to in the text) were allowed to vary during the fitting process.

***Triaxial ellipsoid fitting**:* Non-gelled, lower concentration samples were fitted using the "TriaxialEllipsoidModel" function for non-aligned particles. Fitting was performed on samples within the 1-2 wt% BSA samples where intensity at the low *Q* limit was found to scale linearly with concentration suggesting minimal structure factor contribution. Fitting was found to be robust for 1.5 and 2 wt% BSA with semiaxes parameters (a, b, c) shown in Table S2 and fits shown in Figure S5.

***Ellipsoidal cross section cylinder fitting**:* Fitting was performed using the "EllipticalCylinderModel" for the 1 wt% BSA after heating with fit parameters defined in Table S3 and resulting fit shown in Figure S6.

Correlation length model fitting: In order to fit the azimuthally integrated, background subtracted scattered intensity vs. Q, the shape-independent model function "CorrLength" which makes use of the scattering function described in equation (1) was used. The fitting parameters obtained for the different mass fraction samples are shown in Table S4.

Neonatal rat ventricular myocyte isolation, seeding and culture – All work was carried out under the Animals (Scientific Procedures) Act 1986 and the EU Directive 2010/63/EU (Ethics approval secured by C.M.T). Neonatal rat ventricular myocytes (NRVMs) were isolated from the ventricles of 0-2 day old animals. A GentleMACs neonatal heart dissociation kit (Miltenyi Biotec, Woking, UK) was used to enzymatically digest the ventricles to produce a cell suspension that was pre-plated for 1 hour to remove non-myocytes. The enriched cell isolate was employed for the first set of experiments (cardiomyocyte, CM, -only), while the unselected ventricle digestion was used for the second one. Prior to cell seeding, the substrates (glass coverslips, 4.5 wt% or 9 wt% BSA hydrogels) were coated with 1 μg/ml of human Fibronectin (Sigma-Aldrich) for 1 hour at 37ºC. For validating fibronectin adsorption, coated and pristine samples were stained with an anti-FN polyclonal antibody, followed by an Alexa-594 conjugated secondary antibody. Random pictures from the substrates were taken, and fluorescence quantified with Image J. 500000 cells in 30 µl of M199 with Hank´s salts, 1% v/v L-Glutamine, 1% v/v Penicillin-Streptomycin (all from Sigma-Aldrich), 10% v/v Fetal Calf Serum (Biosera, Nuaille, France) and 0.4 mg/ml vitamin B12 (Sigma-Aldrich) were seeded on the coated substrates, allowed to attach for 30-60 minutes and subsequently supplemented with additional medium. Media was carefully replaced 24 hours after seeding and every other day afterwards.

CM-only experiments: AlamarBlue® and LIVE/DEAD™ viability/cytotoxicity kit (Life technologies, Milton Keynes, UK) test were performed on day 1 following the manufacturer´s instructions. On day 3, substrates were either fixed in 3.7% w/v paraformaldehyde or frozen samples in Trizol. LIVE/DEAD™ stained samples were imaged using an Olympus XI51 inverted microscope.

Ventricular cells experiments: Samples were stained with LIVE/DEAD™ on days 1 and 14, while beating rate was manually counted on days 7 and 14. On days 1, 4, 7 and 14, 3 samples per experiment were frozen for the Picogreen dsDNA quantification assay (Life technologies). Once all had been collected, samples were thawed and digested overnight in a papain (Life technologies) solution. On the next day, they were briefly spin to remove any debris from the supernatant, and the test was carried out following the manufacturer´s instructions. Finally, on day 14, 3 samples were fixed as above and subsequently stained.

RNA extraction, retrotranscription and real time qPCR - Frozen samples in Trizol (Thermo Scientific, Milton Keynes, UK) were slowly defrosted in ice and RNA was extracted following manufacturer´s instructions. Retrotranscription was afterwards performed with QuantiTect Reverse Transcription Kit (Qiagen, Manchester, UK).

Real time qPCR was carried out using the primers listed in Table 1, with SyBr Green Master Mix (Qiagen) using a Rotorgene Q (Qiagen) or Rotorgene 6000 (Corbett Life Science, UK) real thermal cycler. Samples were held at 95°C for 15 minutes to activate the DNA polymerase, then exposed to 40 heat cycles to denature (94°C for 15 seconds), anneal (primer-specific melting temperature for 30 seconds), and extend (72°C for 30 seconds). A melting curve analysis was performed to confirm the length of the PCR products and the specificity of the reaction. All primers were set up to run at efficiencies of 90-100%. Data was normalized against a housekeeping gene (Gapdh) and analyzed using the ΔΔCt method versus the freshly isolated cells (NRVMs) and expressed as relative expression ($2^{-\Delta\Delta Ct}$).

Table S1: List of RT-qPCR primers used.

Gene name	Forward primer	Reverse primer
Gapdh	CAGTGATGGCATGGACTGTG	CAATGCATCCTGCACCAC
Myh7	TTGCTGTTATTGCTGCCATT	ATTGCCAAAGGCCTCCAG
Myh6	TGCTAGCATTGCAGCCATAG	GCCTCCAGAGCAGGGTTAG
Myl2	GGAAGCTCCAACGTGTTCTC	TGTCAATGAAGCCGTCTCTG
Actn2	ATTTCAGGAATGGCCTCAAG	ATTCGCAATCTTGTGGAACC
Tnnt2	AGGCTCTTCATGCCCAACTT	AGTCTGCAGCTCGTTCAGGT
Acta2	GCCGAGATCTCACCGACTAC	GTCCAGAGCGACATAGCACA
Slc8a1	TGGTCTGAAAGATTCCGTGA	GCATACTGGTCCTGGGTAGC
Atp2a2	TTGTAATTCTGCTTATATTGGTAGCC	CTTGCCCATTTCAGGTTCAT
Pln	GCTGAGCTCCCAGACTTCAC	GACAGCAGGCAGCCAAAC
Ryr2	GGCTTCCTCAGTTCCTCCAG	TGAGTGACCTTCAGACATGGA
Gja1	CTCCAAGGAGTTCCACCAAC	CTTGGACCTTGTCCAGAAGC
Gjc1	TGGGAAAGTAACAAACACAGCA	AAGCTCCAACTCATGGTGGT

Immunofluorescence - After fixation, samples were washed in PBS and permeabilized with 1% v/v Tween-20 (Sigma-Aldrich) for 15 minutes at room temperature (RT) and then blocked in 3% v/v donkey serum for 30 minutes at RT. Primary antibodies for Connexin43 raised in mouse (Sigma-Aldrich, C6219, 1:200 dilution), α-actinin raised in rabbit (Sigma-Aldrich, A7811, 1:2000 dilution), cardiac troponin T raised in mouse (Life technologies, MA5-12960, 1:200), Vimentin raised in mouse (Sigma-Aldrich, V6389, 1:25) and Caveolin-1 raised in rabbit (Sigma-Aldrich, C4490, 1:200) were incubated overnight at 4ºC. Samples then were extensively washed in PBS and incubated with the corresponding anti-mouse or anti-rabbit secondary antibodies for 1 hour at RT. Finally, after washing in PBS, nuclei were counterstained with Vectashield with DAPI (Vector Labs, Peterborough, UK), mounted and kept refrigerated protected from light until imaged. For Wheat Germ Agglutinin (WGA, Sigma-Aldrich, L4895) labelling, the samples were fixed, washed in PBS and incubated with 5 µg/ ml WGA solution for 10 minutes at room temperature. After washing in PBS, nuclei were counterstained with Vectashield with DAPI (Vector Labs), mounted and kept refrigerated protected from light until imaged.

Pictures were taken using a Leica SP5 Inverted confocal microscope. Cell area (WGA), density (DAPI) and sarcomere length (alpha-actinin) were quantified using ImageJ.

Statistical analysis - Results are presented as mean ± standard error of the mean (SEM) unless specified otherwise. Statistical analysis was performed using SPSS 22 or GraphPad software. Distributions were assumed normal and differences were analyzed by t-test or ANOVA with a HDS Tukey post-hoc test as required. Differences were considered statistically significant when $p < 0.05$ (*), very significant when $p < 0.01$ (**) and extremely significant when $p < 0.001$ (***).

Atomic force microscopy (AFM) imaging – 1 wt% BSA solution was heated for 5 hours, followed by a 100 fold dilution in water. 20 μl of the diluted solution was placed onto freshly cleaved mica surface that was later used as the substrate for the AFM imaging. After 2 minutes of incubation the mica surface was gently washed in water and dried with a nitrogen stream. An AFM 5500 microscope (Keysight technologies, previously Agilent, Santa Rosa, CA, USA) was used for the imaging in tapping mode. A HQ:NSC15/Al BS tip (μmasch) was used with a tip radius of 8 nm, resonance frequency of 325 kHz and force constant of 40 N/m. The measurements were performed in ambient atmosphere.

Scanning electron microscopy (SEM) imaging – 1 ml of 5 wt% BSA hydrogel was freeze-dried. A small section of the freeze-dried material was taped by carbon tape to a metal stab, and coated with thin layer of Au. A SEM JEOL 5610LV system was used for imaging the surface of the gel, at an operating bias of 18 kV.

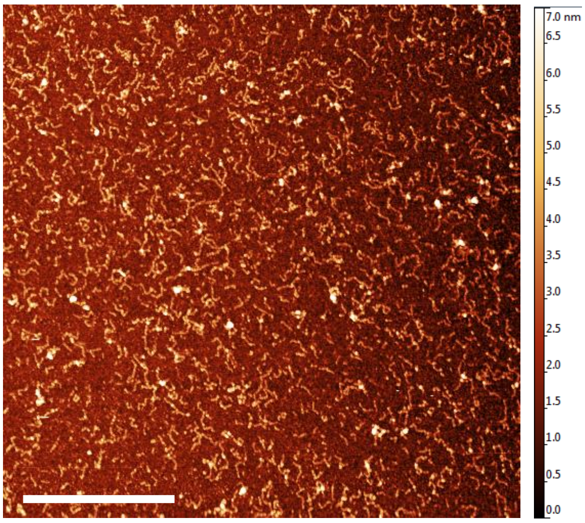

Figure S1. Atomic force microscopy image of BSA fibrils from a 1 wt.% heated BSA solution. The scale bar represents 1 µm. The z-scale of the image is 7 nm.

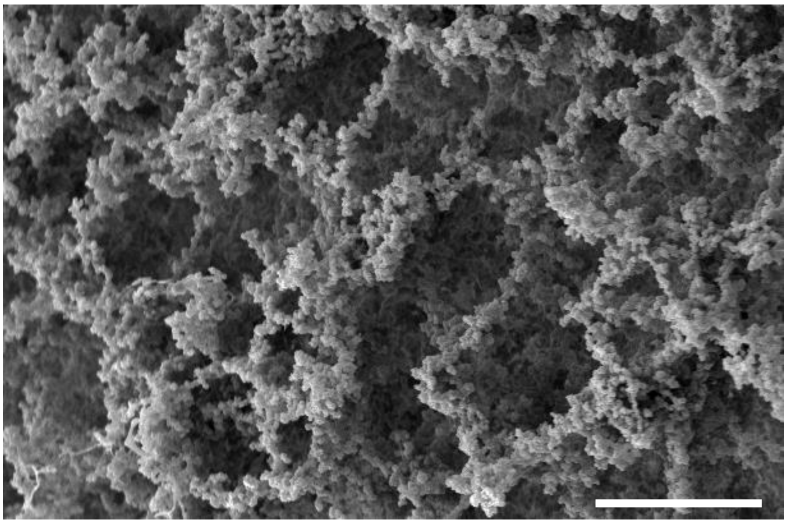

Figure S2. Scanning electron microscopy image of a dried 5 wt.% BSA hydrogel. The scale bar represents 50 µm.

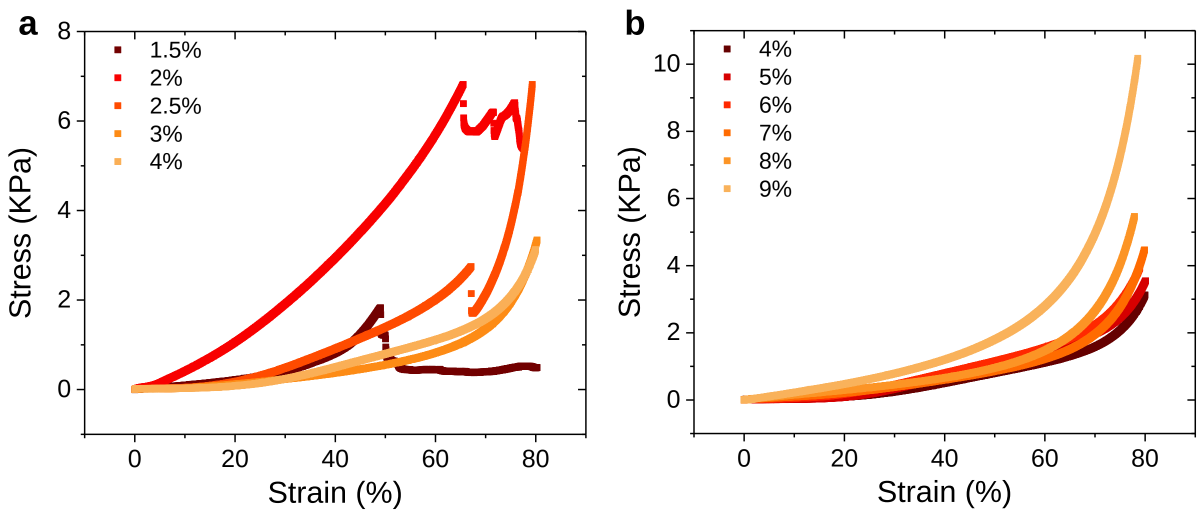

Figure S3. Stress-strain curves spectra for (a) 1.5-4 wt% and (b) 4-9 wt% BSA hydrogels tested under compression.

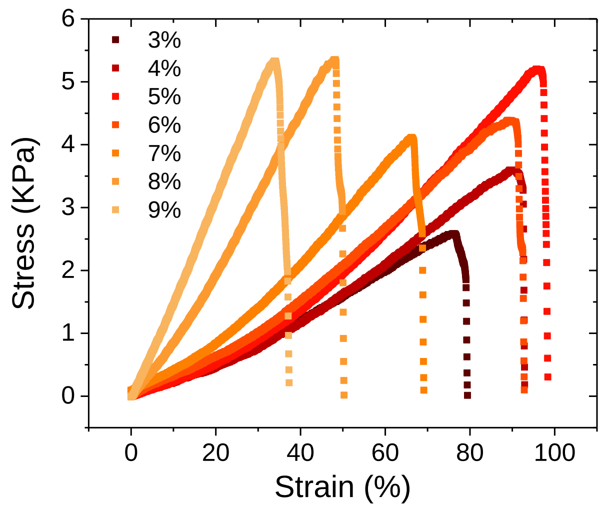

Figure S4. Stress-strain curves spectra for 3-9 wt% BSA hydrogels tested under tension.

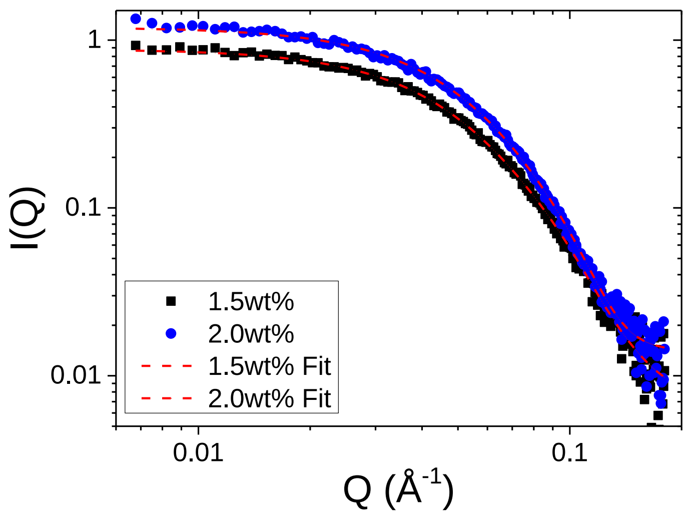

Figure S5. SAXS fitting results for the triaxial ellipsoidal fitting for 1.5 wt.% and 2 wt.% BSA pre-gelled samples.

Table S2. Dimensions obtained for the triaxial ellipsoidal model for the 1.5 wt.% and 2 wt.% BSA pre-gelled samples.

Wt%	χ^2/ *Npts*	*a(nm)*	*b(nm)*	*c(nm)*
1.5	1.14	1.8	3.4	6.8
2.0	1.61	2.2	3.3	6.5

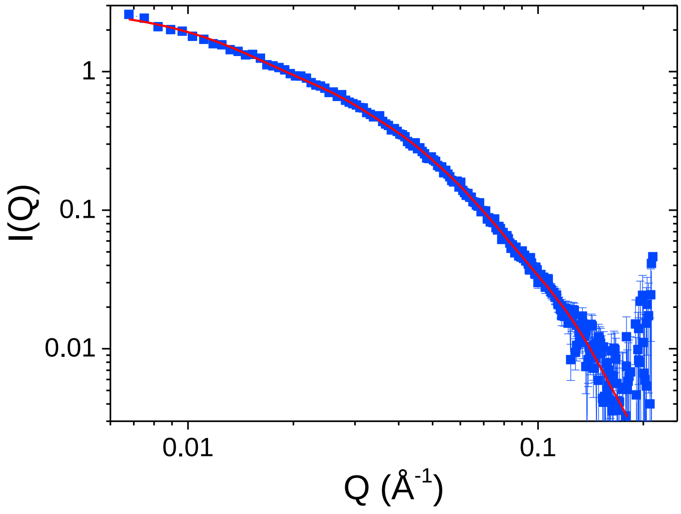

Figure S6. SAXS fitting results for the ellipsoidal cross-section cylinder fitting of the 1 wt% BSA heat treated sample. The high gradient at low *Q* is effectively captured and indicative of elongated structures.

Table S3. Dimensions obtained for the ellipsoidal cross-section cylinder fit for the 1 wt% BSA heat treated sample.

Wt%	χ^2/ *Npts*	R_{minor} *(Å)*	R_{major} *(Å)*	*Length(Å)*
1.0	1.16	14	46	400

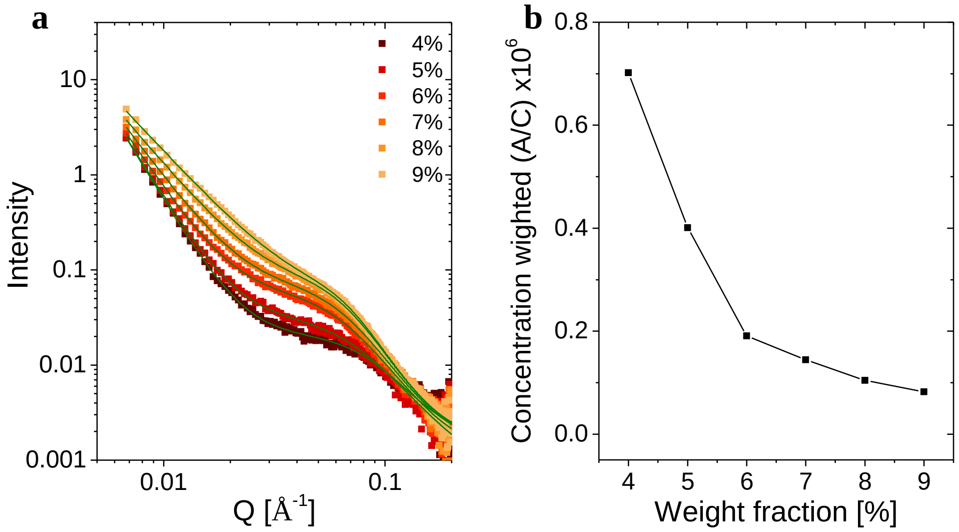

Figure S7. (a) Concentration normalised SAXS intensity for the BSA hydrogels at ≥4 wt.%. The green lines in panel b are the fits according to Eq. 1. (b) The change in the A/C values (see text below) as a function of the BSA hydrogel weight fraction.

Table S4. Fit parameters obtained for the BSA hydrogel samples (except for the 1 wt% BSA sample that does not gel after heating).

Wt.%	χ^2/ *Npts*	*C*	ξ_L *(Å)*	*m*	*A*	*n*
4.0	2.1	0.08	10.4	3.0	2.11 x10^{-7}	3.5
5.0	1.8	0.14	13.2	3.0	2.85 x10^{-7}	3.5
6.0	1.3	0.36	16.9	3.0	4.17 x10^{-7}	3.5
7.0	2.8	0.61	18.6	3.0	6.19 x10^{-7}	3.5
8.0	11.4	1.11	21.2	3.0	9.31 x10^{-7}	3.5
9	35.0	1.85	25.1	3.0	1.37 x10^{-6}	3.5

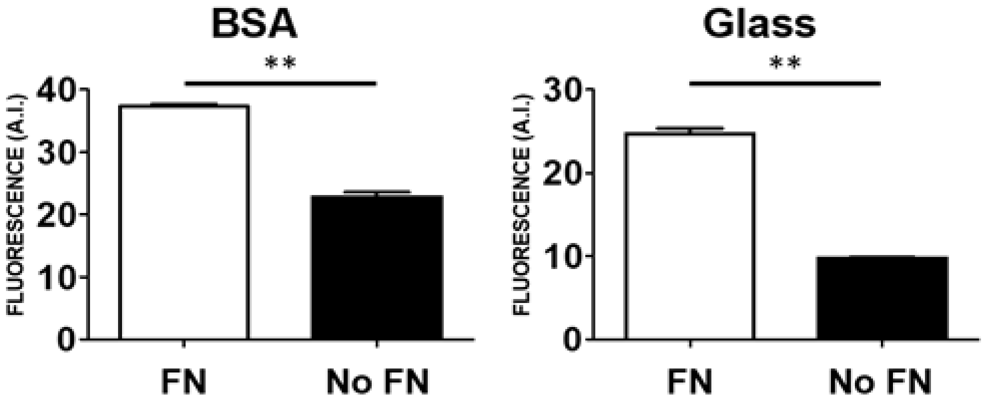

Figure S8. Immunostaining of fibronectin (FN) on 4.5 wt.% BSA hydrogel (left panel) and on glass control (right panel), before (black bars) and after (white bars) FN adsorption. **: $p < 0.01$ vs no FN (t-test). N = 3. All graphs: mean ± SD).

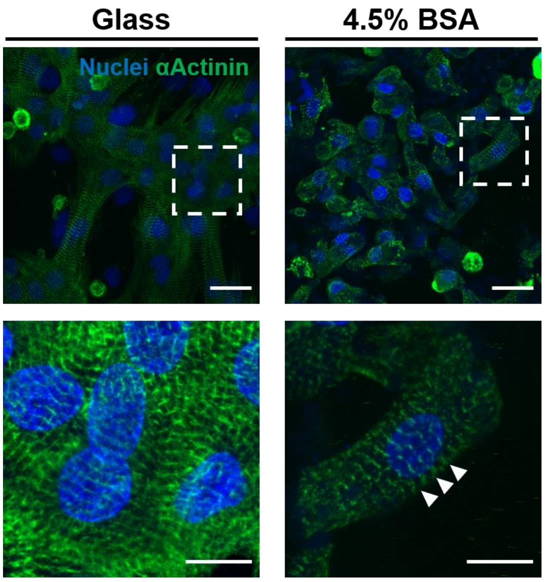

Figure S9. Immunostaining of cardiac α-actinin on CM cultured for 3 days on BSA hydrogels, showing typical striations (arrowheads) on glass (left) and on BSA hydrogel (right). The hydrogel images are of the 4.5 wt.% BSA hydrogel, and there was no significant difference between the 4.5 and 9 wt.% BSA hydrogels. Scale bars: top: 25 µm; bottom: 10 µm.

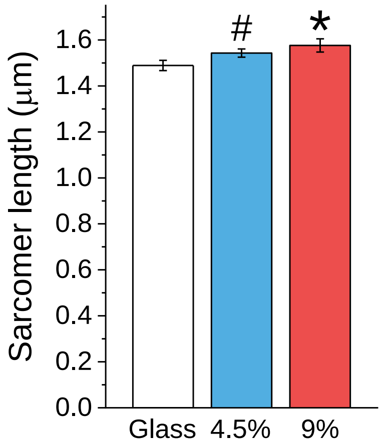

Figure S10. The sarcomere length as quantified from α-actinin stainings. #:$p = 0.06$, *: $p < 0.05$ vs Glass. All graphs: mean ± SEM; n = 3.

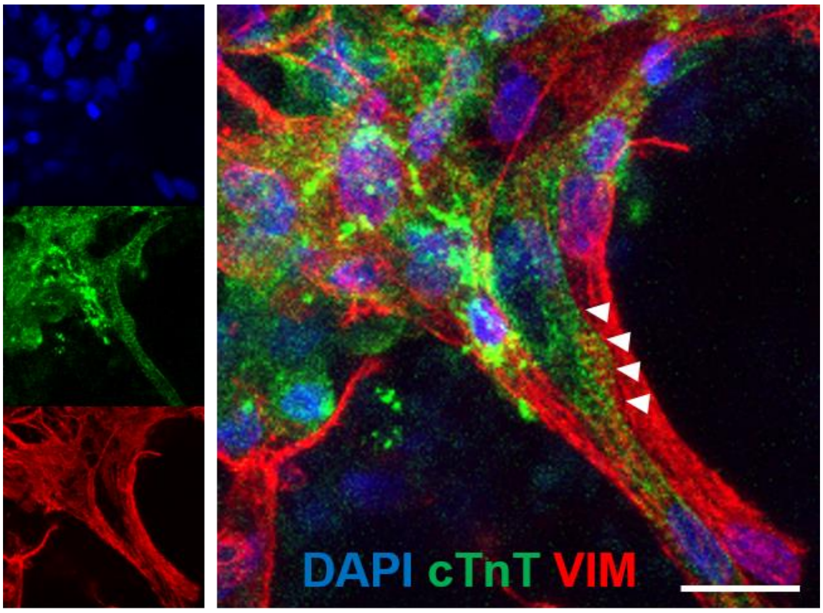

Figure S11. Immunostaining of engineered cardiac tissues showing striations (arrowheads) on cTnT-positive myocytes, which are surrounded by Vim-positive stromal cells. Scale bar: 20 µm.
